# Beam-Membrane Coupled Piezoelectric Micromachined Ultrasonic Transducers with Enhanced Transmitting Sensitivity

**DOI:** 10.3390/mi13030423

**Published:** 2022-03-09

**Authors:** Mengjiao Qu, Xuying Chen, Ke Zhu, Xishan Guo, Jin Xie

**Affiliations:** 1 State Key Laboratory of Fluid Power and Mechatronic Systems, Zhejiang University, Hangzhou 310027, China; 11925060@zju.edu.cn (M.Q.); 11625040@zju.edu.cn (X.C.); 21925035@zju.edu.cn (K.Z.); 2 Department of Biosystems Engineering, Biosensors National Special Lab., Zhejiang University, Hangzhou 310027, China; guoxs@zju.edu.cn; 3 Humanoid Sensing and Perception Center, Zhejiang Lab., Hangzhou 310027, China

**Keywords:** beam-membrane coupled piezoelectric micromachined ultrasonic transducer (BM-PMUT), transmitting sensitivity, MEMS, ultrasound

## Abstract

Piezoelectric micromachined ultrasonic transducers (PMUTs) are a promising alternative to conventional bulk piezoelectric ceramic-based ultrasonic transducers. However, the transmitting sensitivity of the reported PMUTs is far from satisfactory. In this paper, we report a beam-membrane coupled PMUT (BM-PMUT), which enhances the transmitting sensitivity via simultaneously increasing the acoustic emission areas and maintaining the comparable vibration amplitude. Experimental results show that the center and edge transmitting sensitivities of the BM-PMUT are 108.1 and 96 nm/V at 370 kHz, which are 109.9 and 49.6 nm/V at 677 kHz for the traditional PMUT (T-PMUT). Thus, the BM-PMUT realizes piston-like mode shapes and achieves around twofold improvement in the effective acoustic emission area relative to the traditional T-PMUT of the same size. Due to the larger acoustic emission areas and comparable vibration amplitudes, the normalized far-field sound pressure level of the BM-PMUT is 8.5 dB higher than that of the T-PMUT.

## 1. Introduction

Today, micromachined ultrasonic transducers (MUTs) are regarded as a promising alternative to conventional bulk piezoelectric ceramic-based ultrasonic transducers due to the increasing demands for low power consumption, miniaturization, and integration with complementary metal–oxide–semiconductor (CMOS) circuits. Benefiting from the advantages of microelectromechanical systems (MEMS), MUTs have been widely adopted for various applications, such as ultrasonic ranging [1,2], ultrasonic flowmeter [3], medical imaging [4,5,6], and fingerprint identification [7,8], achieving great success. On the basis of the transduction mechanism, MUTs can be divided into two categories: capacitive MUTs (CMUTs) and piezoelectric MUTs (PMUTs) [9]. Compared with CMUTs, PMUTs do not require ultrahigh DC bias (usually >100 V) or an extremely small gap for a satisfactory sensitivity [10,11]. Furthermore, the sensitivity of PMUTs has been enhanced due to the development of the piezoelectric thin-film deposition technique. Hence, PMUTs are a promising solution for ultrasonic applications.

However, PMUTs suffer from low signal-to-noise ratio (SNR) due to the low transmitting sensitivity under limited driving power, which impedes further PMUT-based applications [12,13]. For example, the measurement range for ultrasonic ranging can be increased if the transmitting sensitivity can be optimized. Normally, there are two ways to enhance the transmitting sensitivity of PMUTs: increasing the vibration amplitude or acoustic emission areas. Wang et al. proposed a piston-like PMUT which enhances the transmitting sensitivity via increasing acoustic emission areas [14]. Since the piston-like mode shape could push back and forth more acoustic medium compared with the Gaussian-like mode shape, the piston-like PMUT achieved a 5.3 dB improvement of output sound pressure. Chen et al. put forward a pMUT with V-shaped springs [15]. Combining the advantages of residual stress localization and piston-like mode shape, the output sound pressure of the proposed PMUT could be improved by around 23 dB. Liang et al. utilized the pinning boundary to increasing the vibration amplitude of PMUT [16], and the output sound pressure of the prototyped devices was 3.5 times higher than that of the traditional PMUTs with a clamped boundary. The above studies provide some effective methods for transmitting sensitivity optimization of PMUTs, but the transmitting sensitivity can be further improved since only part of the vibrating diaphragm is utilized. A dual-electrode design can take full advantage of the whole region of PMUTs, and the sensitivity can be effectively optimized. Sammoura et al. designed and fabricated a two-port PMUT which could provide about twice the electromechanical coupling factor and enhance the acoustic power output by about 100% [12]. However, the dual-electrode design needs to be excited by out-of-phase voltage, which increases the complexity of the driving circuits. Furthermore, 3D PMUTs (curved and domed types) also show an improvement in transmitting performance. Wang et al. presented a PMUT modified with a piston diaphragm [17]. Akhbari et al. proposed a curved PMUT [18]. Both cases had higher DC displacements and, thus, generated higher ultrasonic pressure compared to the traditional PMUTs with a similar geometry, but the structures required complex and high-cost fabrication processes, limiting their application.

In this paper, we propose a BM-PMUT with vibrating diaphragm divided into two parts: the outer beam and inner membrane. The two parts of the BM-PMUT vibrate in phase, resulting in piston-like mode shapes. More importantly, the two parts can be excited in phase, thus eliminating the requirement of additional driving circuits. Benefiting from the dual-electrode driving, the BM-PMUT can improve acoustic emission areas while maintaining comparable vibration amplitude, thus achieving an improvement of around 8.5 dB in the normalized far-field sound pressure level (SPL) relative to the T-PMUT. Moreover, other characteristics, including the nonlinear stiffness, sound directivity, and quality factor (Q), are also optimized for the BM-PMUT.

## 2. Materials and Methods

**Figure 1:**
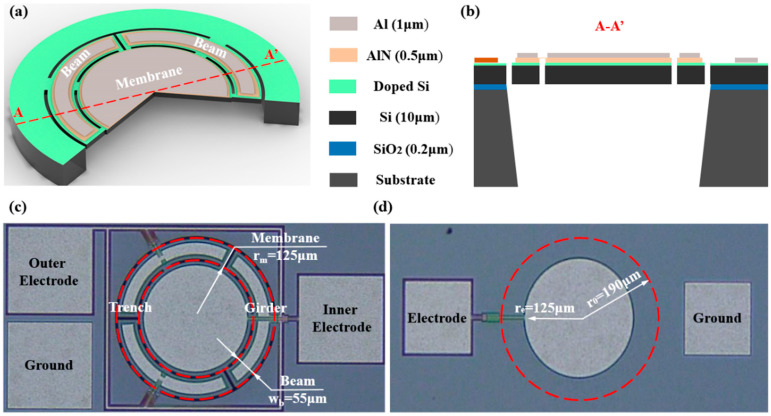
(**a**) Schematic of the BM-PMUT. (**b**) Cross-section structure of A–A’. (**c**) Optical micrograph of the BM-PMUT. (**d**) Optical micrograph of the T-PMUT.

The structure of the BM-PMUT is depicted in Figure 1a,b as a close-up illustration of the cross-section, where the vibrating diaphragm comprises the Al top electrode, AlN piezoelectric layer, doped silicon bottom electrode, and silicon substrate. Figure 1c,d are the optical microscope images of the fabricated BM-PMUT and T-PMUT, respectively. Unlike T-PMUTs which are normally composed of a whole vibrating diaphragm and clamped boundary, the diaphragm of the BM-PMUT is divided into two parts: the outside beams and inside membrane; the boundary is simply supported by three straight girders. When a clamped circular diaphragm (in this work, T-PMUT) of radius *a* vibrates at the first mode, the top inner portion (the inflection point is ~0.7 a from the center [19]) is under tensile stress, and the top outer portion is under compressive stress [20]. To maximize the utilization of the PMUT vibration membrane, the boundary between the beam and membrane (the position of etching groove) is designed at the inflection point. The outside six beams share the same size and are coupled with the inside membrane by six straight girders. The two top electrodes, i.e., the outer electrode and inner electrode, are arranged in the shape of the piezoelectric layer, and they are employed to ensure that the two parts can be excited individually. Since the PMUT usually works in the first-order mode, the electrode designed at the boundary of the compression and tension state can maximize the electromechanical transmission efficiency, according to the stress distribution of first-order vibration. Therefore, the size of the top circular electrode of the T-PMUT has 65% diametral coverage. The two PMUTs share the same dimensions with a diameter of 380 μm. The structure parameters are summarized in Table 1.

**Figure 2:**
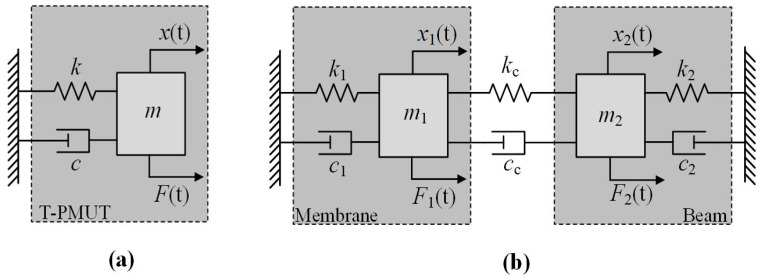
Schematic of the equivalent models for (**a**) T-PMUT and (**b**) BM-PMUT.

The vibration of T-PMUT can be modeled as a one-degree-of-freedom mass–spring–damper system as shown in Figure 2a [21,22], where *m*, *k*, *c*, *x(t)*, *F(t)* represent the effective mass, stiffness, damping (including environment damping and mechanical resistance), displacement, and external forces, respectively. The dynamic equation of this model is

(1) $$m\ddot{x}(t)+c\dot{x}(t)+kx(t)=F(t).$$

The eigenfrequency, i.e., the resonant angle frequency, can be extracted from the free vibration equations for undamped systems, where damping *c* = 0 and external forces *F(t)* = 0. Since the PMUTs are normally operated in a fundamental first-order mode, the first-order resonant frequency of T-PMUT can then be described as

(2) $$f_{1T}=\sqrt{\frac{k}{m}}/2\pi .$$

Similarly, the BM-PMUT can be simplified as a two-degree-of-freedom mass–spring–damper system as shown in Figure 2b; the subscripts 1 and 2 of *m, k*, and *c* represent the inside membrane and outside beam, respectively. *k_c_* is the coupling stiffness and *c_c_* is the coupling damping. When damping *c*_1_ = *c*_2_ = *c_c_* = 0 and external forces *F*_1_*(t)* = *F*_2_*(t)* = 0, the dynamic equations of the model can be simplified to be

(3) $$\left\{ \begin{array}{c} m_{1}{\ddot{x}}_{1}(t)+(k_{1}+k_{c})x_{1}(t)-k_{c}x_{2}(t)=0\\ m_{2}{\ddot{x}}_{2}(t)+(k_{2}+k_{c})x_{2}(t)-k_{c}x_{1}(t)=0 \end{array},\right.$$

where *x*_1_*(t)* and *x*_2_*(t)* represent the displacement of the inside membrane and outside beam, respectively. Consequently, the first-order resonant frequency of the BM-PMUT can be described as

(4) $$f_{1BM}=\sqrt{\left[\frac{1}{2}(\frac{k_{1}+k_{c}}{m_{1}}+\frac{k_{2}+k_{c}}{m_{2}})-\frac{1}{2}\sqrt{{\left(\frac{k_{1}+k_{c}}{m_{1}}-\frac{k_{2}+k_{c}}{m_{2}}\right)}^{2}+4\frac{k_{c}^{2}}{m_{1}m_{2}}}\right]}/2\pi .$$

The first-order resonant frequency of BM-PMUT is mainly determined by the resonant frequency of the beam and membrane. Moreover, it is easy to prove that *f*_1BM_ ≤ *f*_1T_.

## 3. Results

### 3.1. Simulation

**Figure 3:**
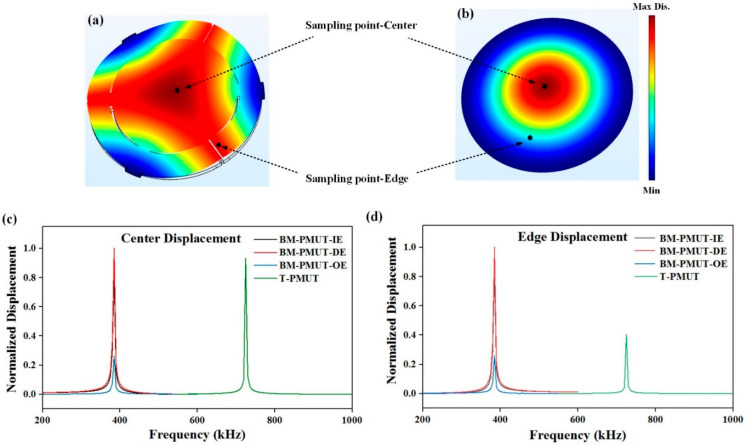
Simulated vibration mode shapes of (**a**) BM-PMUT and (**b**) T-PMUT. Simulated vibration displacement of (**c**) center and (**d**) edge in different structures.

A model of acoustic–piezoelectric interaction was built in COMSOL Multiphysics to investigate the vibration characteristics of the T-PMUT and BM-PMUT. As for the boundary conditions, the T-PMUT was fully clamped, while the BM-PMUT was partly supported at three girders. Figure 3a,b show the simulated first-order vibration mode shapes of the BM-PMUT and T-PMUT; the eigenfrequencies are 385 and 725 kHz, respectively. The vibration mode shape of the BM-PMUT in Figure 3a can be approximated as a fully piston-like mode, since nearly all of the inner membrane and half of the outer beams vibrate with large amplitudes (the yellow and red parts). The effective vibration area is much larger than that of the T-PMUT. For comparison, the T-PMUT vibrates in Gaussian-like mode shapes, and only 22% of the diaphragm maintains large vibration amplitudes, while the value of BM-PMUT is 67%. Hence, the effective acoustic emission area of the BM-PMUT is increased to around three times compared to T-PMUT. Furthermore, there are three types of excitation: inner electrode (IE) excitation, outer electrode (OE) excitation, and dual-electrode excitation (DE) in phase, since the top electrode of BM-PMUT is divided into two parts. Figure 3c,d show the vibration amplitudes of the center and edge in different structures. The BM-PMUT has a higher center displacement due to the decrease in anchor loss resulting from the partly clamped structure. In particular, when the BM-PMUT was excited by the dual electrodes or inner electrode, the edge displacement was much higher than that of the T-PMUT. In general, the beam-membrane coupled structure is designed to enhance the transmitting performance by increasing the vibration amplitude, as well as enlarging the acoustic emission areas.

However, since the boundary of BM-PMUT was changed from fully clamped to partly clamped, the experimental resonant frequency of BM-PMUT was reduced from 725 kHz to 385 kHz, as we can see from the simulated frequency response in Figure 3c,d. Considering the fact that the transmitting sensitivity of a PMUT is normally inversely proportional to its resonant frequency, simulations were further conducted for a fair comparison, as shown next.

**Figure 4:**
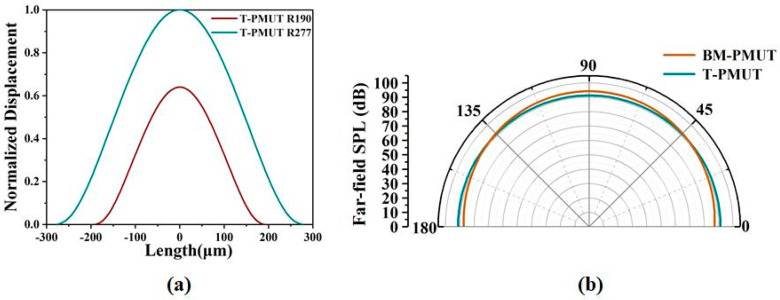
(**a**) Simulated mode shape of the T-PMUT with different radii. (**b**) Simulated far-field SPL of BM-PMUT and T-PMUT.

The SPL was evaluated by the model of acoustic–piezoelectric interaction in COMSOL. The resonant frequency of the T-PMUT was calculated by

(5) $$f_{2}=\sqrt{{\left(3.2/a\right)}^{4}\frac{D}{\mu }}/2\pi ,$$

where *a* is the radius, *D* is the flexural rigidity of the plate, and *μ* is area mass density. The resonant frequency is proportional to 1*/a*^2^. Therefore, the radius of T-PMUT was enlarged from 190 μm to 277 μm to normalize the effect of the resonant frequencies on SPL, considering the error between simulation and theoretical results. Consequently, the transmitting sensitivity of the T-PMUT was improved by around 1.5 times as shown in Figure 4a. Even so, the far-field SPL of the BM-PMUT on the acoustic axis was still 2.5 dB higher than that of the T-PMUT due to the larger acoustic emission area. To be more specific, the acoustic emission area of the BM-PMUT was about 1.4 times higher than that of the T-PMUT with a radius of 277 μm. Furthermore, since the BM-PMUT could save the device areas, i.e., increased fill factor when used in the form of an array, the far-field SPL of BM-PMUT was actually improved by around 8.5 dB when the effect of diaphragm area was normalized. A comparison with previous studies is shown in Table 2. Moreover, the directivity of BM-PMUT was better than that of T-PMUT (as shown in Figure 4b), due to the larger equivalent diameter of the piston-like mode shape [6].

### 3.2. Characterization

**Figure 5:**
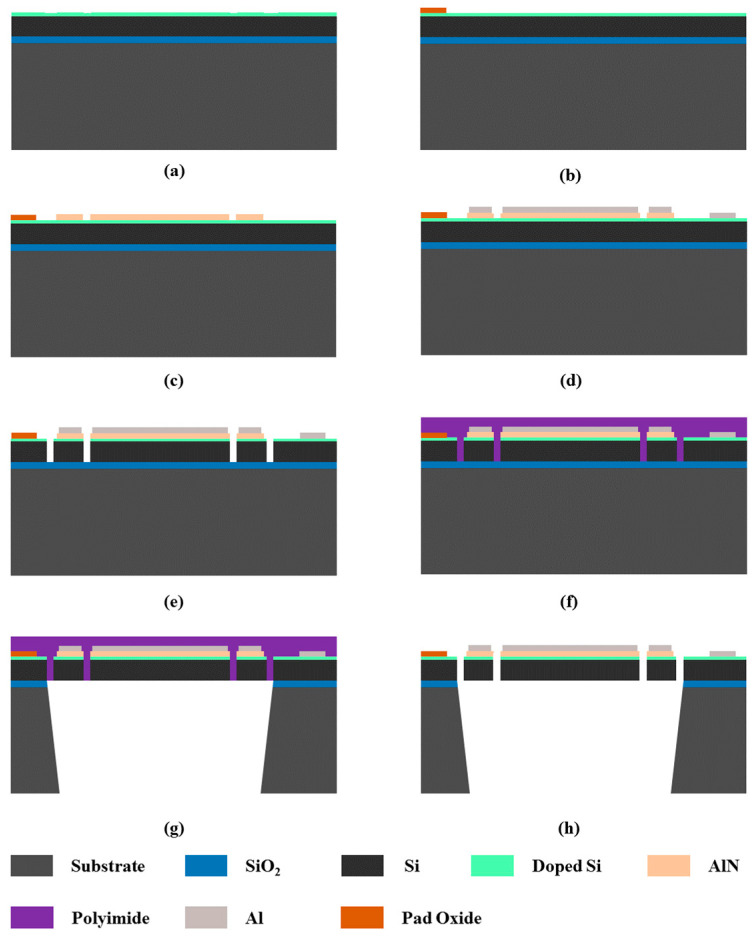
Detailed fabrication process. (**a**) Surface of the structure Si doped. (**b**) Pad oxide layer SiO_2_ thermally grown and wet-etched. (**c**) AlN piezoelectric layer sputtered and patterned. (**d**) Al sputtered and patterned. (**e**) SOI wafer etched. (**f**) Polyimide deposited as a protective layer. (**g**) Backside etching. (**h**) Polyimide removed.

The PMUTs were fabricated by the PiezoMUMPs process from MEMSCAP [10], which starts from an SOI wafer. The detailed fabrication process is shown in Figure 5. The SOI silicon wafer consisted of a 400 nm SiO_2_ insulating layer, 10 μm Si structure layer, and Si substrate, and the surface of the structure silicon was highly p-doped to serve as the bottom electrode (Figure 5a). Next, a 200 nm thick oxide layer SiO_2_ was thermally grown on its surface and wet-etched using a photoresist mask (Figure 5b). Then, a 500 nm thick AlN piezoelectric layer and 1 μm Al were sputtered and patterned (Figure 5c,d). Then, the device layer of SOI was etched by DRIE (deep reactive ion etching) to define the structure on the diaphragm of the BM-PMUT (Figure 5e). A layer of polyimide was deposited as a protective layer (Figure 5f); then, the handle layer of the SOI and the buried oxide were etched by DRIE and wet etching, respectively, on the back side (Figure 5g). Finally, the polyimide layer was removed.

**Figure 6:**
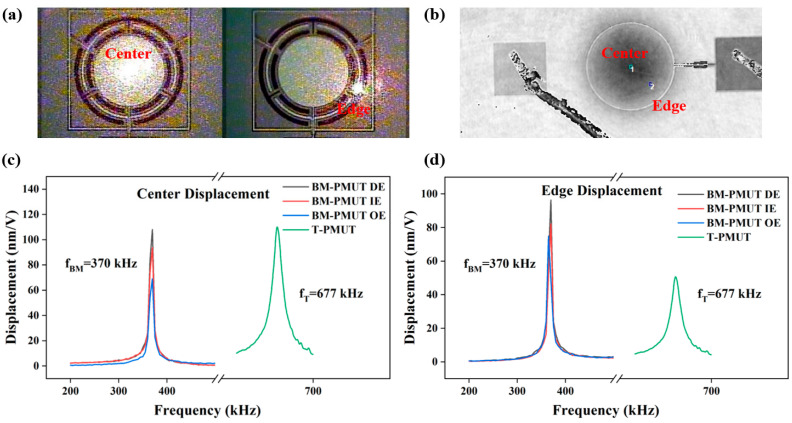
Sampling points on the center and edge selected for (**a**) BM-PMUT and (**b**) T-PMUT. Measured resonant frequencies and (**c**) center and (**d**) edge displacement of the BM-PMUT and T-PMUT.

**Figure 7:**
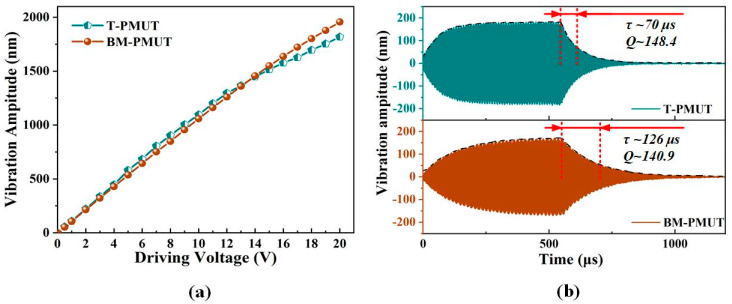
(**a**) The vibration amplitude at the resonant frequencies for different driving voltages of T-PMUT and BM-PMUT. (**b**) The time-domain vibration characteristics at resonant frequencies for T-PMUT and BM-PMUT.

The vibration amplitudes of the BM-PMUT were measured through a laser Doppler vibrometer (LDV, NLV 2500, Polytec). The position of the laser point on the center or edge of the BM-PMUT device was adjusted carefully according to the image in the software using a high-precision positioner, as shown in Figure 6a. Due to the limited measurable frequency range of LDV, the vibration characteristics of the T-PMUT were investigated by a digital holographic MEMS analyzer (Lyncee Tec. DHM-R2100). The sampling points on the center and edge of the T-PMUT are shown in Figure 6b. The measured displacement/voltage result of the BM-PMUT was exported directly from the software, while it was computed by subtracting the upper and lower envelopes of the vibration curve for the T-PMUT, as shown in Figure 6c,d. The two sampling points selected for each PMUT are consistent with those in Figure 3a,b. Moreover, three driving modes, inner electrode, outer electrode, and dual electrodes, were evaluated for the BM-PMUT. The resonant frequency deviation was mainly caused by manufacturing errors. The experimental results were consistent with the simulation results. For the BM-PMUT, the vibration amplitude of the edge (without anchor) was slightly less than that of the center; however, for the T-PMUT, the vibration amplitude of the edge was far less than that of the center. Specifically, the center and edge vibration amplitudes of BM-PMUT are 93 and 82 nm when only the inner electrode was excited, corresponding to 109.9 and 49.6 nm for the T-PMUT. The relatively lower center vibration amplitude of the BM-PMUT is mainly due to the additional air damping caused by the larger effective vibration area and etching trenches [6]. The center vibration amplitude of the BM-PMUT could be improved to 108.1 nm when the dual electrodes were excited in phase, which was comparable to the T-PMUT. Meanwhile, the edge vibration amplitude of the BM-PMUT was 96 nm, which is nearly two times of the T-PMUT. Furthermore, Figure 7a compares the center vibration amplitude as a function of the driving voltage (at respective resonant frequencies) for both the BM-PMUT and the T-PMUT. Although the T-PMUT attained higher vibration amplitude at low voltage, the heavy nonlinearity decelerated the increase in vibration amplitude. Hence, the vibration amplitude of the BM-PMUT exceeded that of the T-PMUT for voltages above 13 V. The nonlinear stiffness characteristic is a common phenomenon observed in MEMS structures with a fully clamped boundary, which is caused by the displacement-induced tensile stress occurring upon large deflection of the diaphragm [23]. However, the partly clamped boundary of the BM-PMUT mitigated the effect of displacement-induced tensile stress in the diaphragm. Figure 7b shows the time-domain vibration characteristics at respective resonant frequencies for both T-PMUT and BM-PMUT. The black dotted lines are the envelopes corresponding to each waveform. The measured ring-down time was 70 μs and 126 μs for the T-PMUT and BM-PMUT, respectively, and the quality factor *Q* was calculated as follows:

(6) Q=πfτ

where the time constant *τ* is the ring-down time for the vibration amplitude to decay to 1*/e* of the initial value, and it is equal to 2*M_m_/R_m_* (*M_m_* is the equivalent mass, and *R_m_* is the equivalent damping). Consequently, the corresponding *Q* was 148.8 and 140.9. The slightly lower *Q* of the BM-PMUT was the comprehensive result of the additional air damping as aforementioned, optimized thermoelastic damping [24], and anchor loss. A PMUT with lower *Q* is favorable for ultrasonic pulse applications.

## 4. Conclusions

To sum up, an AlN-based beam-membrane coupled piezoelectric micromachined ultrasonic transducer was fabricated and characterized. The BM-PMUT can realize a fully piston-like mode shape since the outer beam and inner membrane of the BM-PMUT vibrate in phase. Compared with the T-PMUT of the same size, the BM-PMUT had the same center displacement but nearly three times higher edge displacement under dual-electrode excitation, contributing to an improvement of around 3.5 times in the effective acoustic emission areas. Simulation results show that the normalized far-field SPL could be improved by around 8.5 dB. Moreover, the BM-PMUT demonstrated an improved linear displacement limit for high driving voltages, better sound directivity, and lower Q compared to the T-PMUT. The proposed BM-PMUT is a promising alternative in many applications of ultrasonic transducers.

## Figures and Tables

**Table 1:** Summary of the structure parameters.

Symbol	Value	Description
r_0_	190 μm	Cavity radius
r_e_	125 μm	Top electrode radius of T-PMUT
r_m_	125 μm	Inside membrane radius of BM-PMUT
w_b_	55 μm	Outside beam width of BM-PMUT
w_t_	5 μm	Trench width of BM-PMUT
w_g_	5 μm	Girder width of BM-PMUT
h_Al_	1 μm	Thickness of top electrode
h_AlN_	500 nm	Thickness of piezoelectric layer
h_Si_	10 μm	Thickness of Si structure layer
h_SiO2_	200 nm	Thickness of SiO_2_ insulating layer

**Table 2:** Comparison with previous works.

	This Work	Ref. [11]	Ref. [15]
Method	Beam-membrane structure	Etching holes	Pinned boundary
Optimized objects	Acoustic emission area	Acoustic emission area	Vibration amplitude
Improvement in SPL (dB)	8.5	5.3	4.65 *

* Computative results.
